# Electrical and Magnetic Transport Properties of Co_2_VGa Half-Metallic Heusler Alloy

**DOI:** 10.3390/ma15176138

**Published:** 2022-09-04

**Authors:** Litao Yu, Zhe Li, Jiajun Zhu, Hongwei Liu, Yuanlei Zhang, Yiming Cao, Kun Xu, Yongsheng Liu

**Affiliations:** 1 Department of Physics, Shanghai University of Electric Power, Shanghai 200090, China; 2 Center for Magnetic Materials and Devices, College of Physics and Electronic Engineering, Qujing Normal University, Qujing 655011, China; 3 Yunnan Zhongruans Liquid Metal Technology Co., Ltd., Qujing 655400, China; 4 Key Laboratory for Anisotropy and Texture of Materials (Ministry of Education), School of Material Science and Engineering, Northeastern University, Shenyang 110819, China

**Keywords:** Heusler alloy, half-metallic ferromagnetism, anomalous Hall effect

## Abstract

This study performed a systematic experimental investigation into the structural, magnetic, and transport properties of the Co_2_VGa Heusler alloy, which was theoretically predicted to exhibit half-metallic ferromagnetism. It has been experimentally found that the studied alloy has a relatively high-ordered *L*2_1_ cubic structure at room temperature and orders ferromagnetically below ~350 K. Interestingly, by fitting the electric transport data with the properly governing equations in two different temperature regions, the two-magnon scattering process (the $T^{9/2}$ dependence) appears in the temperature range from 30 to 75 K. Moreover, the magnetoresistance effect changes from a negative value to a positive value when the temperature is below 100 K. Such experimental findings provide indirect evidence that the half-metallic nature of this alloy is retained only when the temperature is below 100 K. On the other hand, the magnetic transport measurements indicate that the anomalous Hall coefficient of this alloy increases when the temperature increases and reaches a relatively high value (~8.3 μΩ·cm/T) at 300 K due to its lower saturated magnetization. By analyzing the anomalous Hall resistivity scale with the longitudinal resistivity, it was also found that the anomalous Hall effect can be ascribed to the combined effect of extrinsic skew scattering and intrinsic Berry curvature, but the latter contribution plays a dominant role.

## 1. Introduction

Since the concept of half-metallic ferromagnetism was proposed by de Groot et al. for the NiMnSb half-Heusler alloy [1], half-metallic ferromagnets have attracted considerable attention in the fields of physics and material science owing to their exciting potential for application in spin electronics [2]. Henceforth, half-metallic ferromagnetism has been developed in a variety of materials, such as full Heusler alloys [3], binary oxides [4], antiferromagnets [5], perovskite manganite oxides [6], and spinel-type compounds [7]. A common feature of them is that one spin channel shows a metallic character, whereas the other channel shows an insulative character. Such a unique electronic band structure results in a complete spin polarization of 100% at the Fermi level. However, it is remarkable that Co_2_-based full Heusler alloys have become the most promising half-metallic ferromagnets for practical applications when compared with the others mentioned above, due to their high Curie temperature, large magnetization, and relatively broad band gaps [8]. 

Among the Co_2_-based half-metallic ferromagnets, the stoichiometric Co_2_VGa alloy seems to be a prospective candidate. Over the past decade, both theoretical and experimental investigations have been performed to comprehend the crystalline structure, electronic band structure, and magnetic property of this alloy [9,10,11,12]. It has been found that the Co_2_VGa alloy possesses a *L*2_1_-type cubic structure in a wide temperature range and its energy gap produced by a half-metallic band is determined to be ~0.2 eV at the Fermi level. In the meantime, the total magnetic moment (~2.0 μ_B_) of this alloy was also found to follow the generalized Slater–Pauling rule ($m=N_{V}-24$, where $N_{V}$ is the number of accumulated valence electrons in the unit cell), which is mainly contributed by the Co atom, whereas the V atom has a magnetic moment of only ~0.2 μ_B_. More importantly, the total magnetic moment of the Co_2_VGa alloy at 5 K was found to be independent of pressure based on magnetic measurements under high pressure [9]. This is the sole piece of experimental evidence used to prove that it has a half-metallic band structure at extremely low temperatures until now. During the past several decades, the investigation of electrical transport in half-metallic ferromagnets has been the subject of intense interest, because their transport behavior could depend on specific electron–magnon interactions [13,14,15,16]. These studies also indicate that an in-depth exploration of the resistivity change with temperature can offer more information to experimentally confirm the half-metallic nature of the Co_2_VGa alloy.

In addition to half-metallic ferromagnetism, some Co_2_-based full Heusler alloys also exhibit a large anomalous Hall effect (AHE) owing to their particular electronic band structures, such as Co_2_MnAl [17] and Co_2_MnGa [18] alloys. It is well known that two possible mechanisms exist which have been put forward to explain the origin of the AHE. One is the extrinsic mechanism related to skew scattering or the side-jump [19,20], while the other is the intrinsic mechanism related to the Berry curvature, depending on the electronic band structure of the materials [21]. Very recently, Shukla et al. separated the relative contribution of the extrinsic and intrinsic components to the anomalous Hall conductivity (AHC) in Co_2_FeGe and Co_2_FeAl alloys, respectively, and found that the AHC of these compounds at 50 K emerged from the intrinsic Berry curvature that accounts for ~70% of the total contribution [22,23]. Nevertheless, there is only a small amount of information available about the origin of the AHE on the Heusler alloy Co_2_VGa with high spin polarization. Therefore, it is meaningful to further elucidate the origin of the AHE for the Co_2_VGa alloy based on combining magnetic transport measurements with existing viewpoints.

In this work, the electrical and magnetic transport properties of the polycrystalline Co_2_VGa Heusler alloy were systematically investigated. A detailed analysis of both the longitudinal resistivity and magnetoresistance effect supports the suggestion that the half-metallic nature of this alloy is retained only up to 100 K. Moreover, the analysis of magnetic transport data gives an experimental value of the AHC being ~91 S/cm at 60 K with an intrinsic contribution of ~57 S/cm, which demonstrates that the AHE of this alloy is mainly due to the Berry curvature.

## 2. Materials and Methods

The polycrystalline Co_2_VGa alloy with nominal composition was fabricated by arc melting in a high-purity Ar atmosphere. The ingot obtained was annealed in an evacuated quartz tube for 24 h at 1473 K and subsequently quenched in ice water. The real composition corresponded to Co_1.981_V_0.995_Ga_1.024_ which was semi-quantitatively analyzed by energy dispersive X-ray spectroscopy (EDS, ProX, Phenom, Eindhoven, the Netherlands). The crystal structure at room temperature was characterized using powder X-ray diffraction (XRD, Ultima-IV, Rigaku, Tokyo, Japan) and refined using the Rietveld refinement approach [24]. Prior to the XRD measurement, a part of the bulk specimen was manually ground into the powder with a grain size of ~90 μm, which was further annealed at 873 K for 6 h. Heat flow data were collected using a differential scanning calorimeter (DSC, Q2000, TA Instruments, New Castle, PA, USA). The magnetization data were gathered using a vibrating sample magnetometer (VSM, Versalab, Quantum Design, San Diego, CA, USA). The specimens were cut into the dimensions of 1 × 1 × 5 mm^3^ and 6 × 6 × 0.3 mm^3^ for electrical resistivity and Hall resistivity measurements, respectively, which were performed on a Versalab equipped with an electrical transport option based on the four leads method. The raw Hall resistivity was measured along the positive ($\rho_{xy\left(+H\right)}$) and negative fields ($(\rho_{xy\left(-H\right)}$). To eliminate the effect of longitudinal resistivity, the real Hall resistivity was obtained using the formula of $(\rho_{xy\left(+H\right)}-\rho_{xy\left(-H\right)})/2$. In addition, the electrical resistivity in the temperature range of 5–50 K was recorded using a physical property measurement system (PPMS, DynaCool, Quantum Design, San Diego, CA, USA).

## 3. Results and Discussion

**Figure 1:**
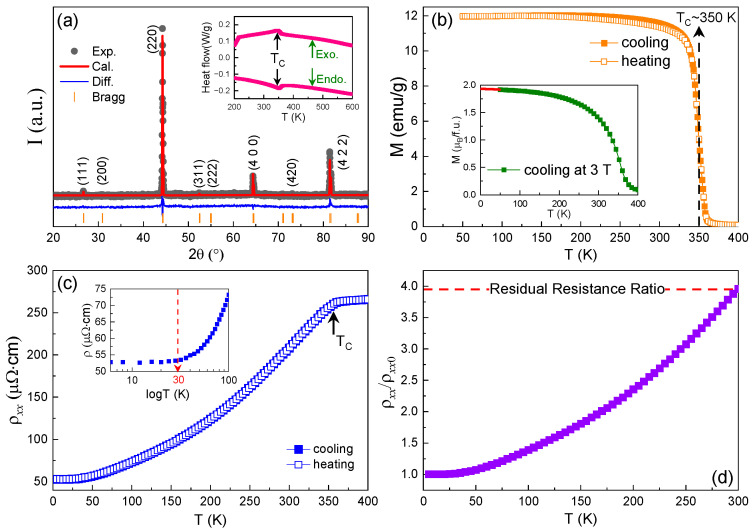
(**a**) The powder X-ray diffraction (XRD) measured at room temperature. The inset shows the heat flow data on cooling and heating modes. (**b**) Thermomagnetic curves measured at 0.05 T. The inset shows the total magnetic moment determined by the linear extrapolation thermomagnetic curve of 3 T to zero Kelvin. (**c**) The temperature dependence of longitudinal resistivity $\rho_{xx}\left(T\right)$ measured at zero magnetic field. The inset shows the $\rho_{xx}\left(T\right)$ curve on a logarithmic scale in a low temperature region. (**d**) The $\rho_{xx}/\rho_{xx0}$ versus temperature curve in the temperature range from 5 K to 300 K ($\rho_{xx0}$ represents the residual resistivity). The red dashed line denotes the residual resistance ratio (RRR) which is determined by the formula of $\mathrm{RRR}=\rho_{xx\;\left(300\;K\right)}/\rho_{xx0}$.

Figure 1a shows the XRD pattern of the Co_2_VGa alloy at room temperature, which was refined using the *L*2_1_ ordered cubic structure with the space group *Fm*$\overset{-}{3}$*m*. From the diffraction spectrometer, one can see that the diffraction peaks presented in the experimental data (depicted as black hollow dots) correspond well those presented in the calculated data (depicted as a red solid line). It is worthwhile to mention that the intensity of (200) the superlattice reflection is unable to be distinguished because its relative intensity is four orders lower than that of (200) the diffraction peak. This can be explained by the fact that the scattering factors for the Co, V, and Ga atoms in the lattice are very close, rather than caused by the chemical disorder. Similar results were also reported in the Co_50_V_35_Ga_14_Ni_1_ quaternary Heusler alloy [25]. As evidenced from the inset of Figure 1a, no signals related to the structure or atomic order–disorder transitions can be detected in the endothermic and exothermic curves, except for the existence of a small peak at the $T_{C}$ (the Curie temperature). The value of the $T_{C}$ determined from the heat flow data is ~350 K, which corresponds exactly to the occurrence of ferromagnetic transition. More details are reflected by the low-field thermomagnetic curves measured in Figure 1b. These findings suggest that a relatively high-ordered *L*2_1_ structure can be expected in the studied alloy, which agrees with the atomic configuration of the Co_2_VGa alloy confirmed by the neutron diffraction technique [26]. Moreover, the lattice parameter of this alloy was found to be 5.7834 Å, which approaches the value reported previously [9]. The total magnetic moment of ~1.93 μ_B_ was estimated from the linear extrapolation of a high-field thermomagnetic curve to zero Kelvin (see inset of Figure 1b). This value is in good agreement with that predicted by the first-principles calculation [11,27] and the Slater–Pauling rule [28]. Figure 1c shows the temperature dependence of the longitudinal resistivity $\rho_{xx}\left(T\right)$ at zero magnetic field. In the process of cooling and heating, both $\rho_{xx}\left(T\right)$ curves coincide perfectly and exhibit a typical metallic behavior. In the paramagnetic state (above the $T_{C}$), the $\rho_{xx}$ decreases mildly with decreasing temperature and presents a linear feature, which can be attributed to the weakening of electron–phonon scattering. Nevertheless, in the ferromagnetic state (below the $T_{C}$), the resistivity decreases strikingly and presents a nonlinear feature. A similar phenomenon was usually observed in Co_2_-based half-metallic Heusler alloys [15,16], which can be ascribed to the contributions of different scattering processes. To obtain a better understanding of the evolution of the resistivity at low temperatures, the $\rho_{xx}\left(T\right)$ curve was plotted on a logarithmic scale in the range of 5−100 K, as shown in the inset of Figure 1c. A relatively wide platform can be observed in the $\rho_{xx}\left(T\right)$ curve when the temperature is below 30 K, suggesting that the value of the $\rho_{xx}$ in this region is nearly independent of temperature. In this case, the value of the longitudinal residual resistivity ($\rho_{xx0}$) caused by impurities and defects can be obtained by linearly extrapolating this curve from 30 to 0 K, and the variation in the $\rho_{xx}/\rho_{xx0}$ with temperature is illustrated in Figure 1d. It is obvious that the residual resistance ratio (RRR) of the sample equals ~3.9, which is comparable to that which was reported for the Co_2_MnSi Heusler alloy with a highly atomic order [29] and is significantly larger than those reported in other Co_2_-based Heusler alloys with a higher degree of anti-site disorder [15,16,23].

**Figure 2:**
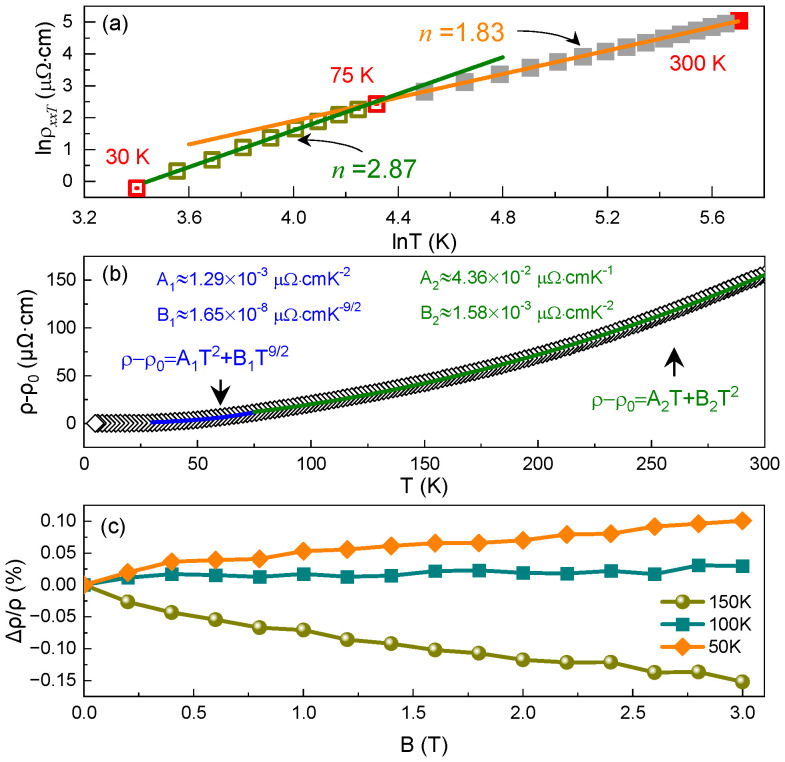
(**a**) The $\rho_{xxT}$ versus T plotted on a double Napierian logarithm scale. Solid black and orange lines are linear fitting in the range of 30−75 K and 75−300 K, respectively. (**b**) The $\rho_{xxT}$ as a function of temperature. Solid blue and olive lines are fitting results in the range of 30−300 K by using Equations (1) and (2), respectively. (**c**) Magnetic field dependence of the magnetoresistance at selected temperatures.

For traditional ferromagnetic materials, several scattering mechanisms can be employed to explain the variation in the $\rho_{xx}$ depending on temperature, such as electron–phonon interaction, electron–electron interaction, and electron–magnon interaction, including single-magnon scattering or two-magnon scattering. In fact, the contribution of electron–phonon scattering to the $\rho_{xx}$ is negligible at low temperatures compared to that of the other scattering processes [30]. In the case of true half-metals, however, it is necessary to emphasize that single-magnon scattering is possibility forbidden owing to the existence of a gap in the density of states (DOS) of the minority sub-band [31]. Therefore, two-magnon scattering is often regarded as an intrinsic signature in half-metallic ferromagnetic Heusler alloys, whose resistivity follows the $T^{9/2}$ dependence at low temperatures and the $T^{7/2}$ dependence at high temperatures [32]. Taking into account this fact, the $\rho_{xxT}$ (i.e., $\rho_{xx}-\rho_{xx0}$) versus T curve was drawn on a double Napierian logarithmic scale, as shown in Figure 2a. Two different linear relationships between the $\rho_{xxT}$ and the T can be distinguished in this coordinate system, which are divided by a critical temperature of ~75 K. This indicates that the longitudinal resistivity presented in these two temperature regions should correspond to different scattering mechanisms. The values of the exponent n derived from the slopes of these two straight lines are ~2.87 below 75 K and ~1.83 above 75 K, respectively. Apparently, a relatively low value of the exponent n obtained in the range of 75−300 K also hints that the $T^{7/2}$ term linked to two-magnon scattering could not exist in here.

Based on the aforementioned analysis, the $\rho_{xxT}$ of the Co_2_VGa alloy as a function of temperature is fitted using two suitable governing equations for low- and high-temperature regions, as shown in Figure 2b. First, we fit the longitudinal resistivity curve from 30 to 75 K using the following equation:

(1) $$\rho_{xxT}=A_{1}T^{2}+B_{1}T^{9/2}$$

where $A_{1}$ and $B_{1}$ signify the fitting coefficients, which are recorded in Figure 2b; the $T^{2}$ and $T^{9/2}$ terms correspond to the electron–electron and two magnon scattering processes, respectively. From Figure 2b, it can be observed that the fitting curve agrees well with the experimental data in this temperature region. The presence of the $T^{9/2}$ term provides a signature of the half-metallic nature of the studied alloy. In the temperature range of 75−300 K, the resistivity curve region is found to fit well with the following equation:

(2) $$\rho_{xxT}=A_{2}T+B_{2}T^{2}$$

where $A_{2}$ and $B_{2}$ denote the fitting coefficients shown in Figure 2b. The fitting result indicates that the scattering processes of this alloy could be governed by single-magnon interaction and electron–phonon interaction (the T dependence) in the temperature range from 75 to 300 K. Figure 2c further presents the magnetic field dependence of the magnetoresistance at selected temperatures. With an increase in the magnetic field, the longitudinal resistivity decreases when the temperature is above 100 K, performing a negative magnetoresistance effect. This can be explained by the alignment of the magnetic moments which lowers the single-magnon scattering. If the temperature is below 100 K, however, a small positive magnetoresistance can be observed, because only one spin state is exhibited at the Fermi level. The increase in longitudinal resistivity with an increasing magnetic field can probably be understood by the enhancement of the exchange of the peripheral electron between Co atoms, which strengthens the two-magnon scattering. Therefore, we can conclude that the Co_2_Vga alloy retains its half-metallic character when the temperature is below 100 K. Analogous results have also been reported for other Co_2_-based half-metallic compounds [15,16].

**Figure 3:**
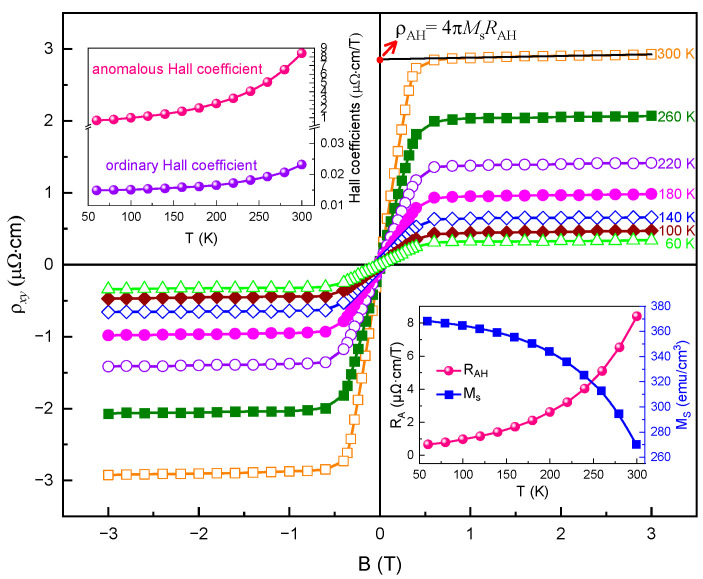
Magnetic field dependence of total Hall resistivity ($\rho_{xy}$) at various temperatures. The black solid line shows the linear fitting data for an ordinary Hall part which is used to extract the ordinary and anomalous Hall coefficients. The left top inset shows the temperature dependence of the ordinary ($R_{0}$) and anomalous Hall coefficients ($R_{AH}$). The right bottom inset shows the comparison between temperature dependence of $R_{AH}$ and saturated magnetization ($M_{s}$).

Next, we focus on studying the AHE of the Co_2_Vga alloy based on a detailed magnetic transport measurement carried out on its ferromagnetic state. Figure 3 shows the change in the total Hall resistivity ($\rho_{xy}$) with the magnetic field (B), which was measured in the range of 60−300 K up to a magnetic field of 3 T. Noticeably, the value of $\rho_{xy}$ is almost two orders of magnitude lower than that of the $\rho_{xx}$, proving that longitudinal resistivity does not contribute to the Hall resistivity. In the low field region, the $\rho_{xy}$ rapidly increases until the field is close to 0.5 T, indicating the occurrence of the AHE. When the field surpasses 0.5 T, the $\rho_{xy}$ nearly reaches a saturated value and reveals a monotonically slight increment, reflecting a signature of the ordinary Hall effect. Normally, the total $\rho_{xy}$ can be expressed by the following empirical equation [21]:

(3) $$\rho_{xy}=R_{0}B+4\pi M_{s}R_{AH}$$

where $R_{0}$ and $R_{AH}$ denote the ordinary and anomalous Hall coefficients, respectively, while $M_{s}$ stands for the saturated magnetization. By linearly fitting the ordinary Hall data at high fields, both the $R_{0}$ and $R_{AH}$ ($R_{AH}=\rho_{AH}/4\pi M_{s}$) can be deduced from the slope and intercept of the saturated part of the $\rho_{xy}(B)$ curve (depicted as the black solid line in Figure 3). The obtained values of $R_{0}$ and $R_{AH}$ as functions of temperature are shown in the left top inset of Figure 3. One can see that the $R_{0}$ is very small and faintly increases as the temperature increases. When compared with the $R_{0}$, the $R_{AH}$ improved approximately by two orders of magnitude. At low temperatures, the $R_{AH}$ increased slowly with the increase in temperature, while when the T>200 K, it reveals a substantial enhancement. Actually, a sharp change in the $R_{AH}$ around 100 K was also reported in the Co_2_FeSi alloy, because the disappearance of the half-metallicity may affect the Berry phase or the side-jump scattering contribution to its AHE [33]. On the contrary, by comparing the temperature dependence of the $R_{AH}$ and $M_{s}$ for the studied alloy, such an increase in the $R_{AH}$ can be attributed to an evident variation in the $M_{s}$ in the temperature range from 200 to 300 K, as shown in the right bottom inset of Figure 3. This result suggests that the direct correlation between the AHE and half-metallicity cannot be found in our studied alloy. At the same time, it is noticeable that the $R_{AH}$ of the studied alloy arrives ~8.3 μΩ·cm/T at 300 K, which is at least dozens of times larger than that reported for the Co_2_FeSi monocrystal [33] and Co_2_FeAl polycrystal [23] alloys, as well as greatly exceeding that reported in the Ni-Mn-based metamagnetic Heusler alloy near room temperature [34]. Such a relatively high $R_{AH}$ exhibited by the studied alloy can be interpreted as the fact that it has a lower $M_{s}$. Additionally, according to the one-carrier model, the carrier concentration (n) and mobility of carriers (μ) can be acquired from the relations of $n=1/qR_{0}$ and $\mu =1/qn\rho_{xx}$, respectively, where q is the elementary charge. The calculated n and μ were ~2.7 × 10^22^/cm^3^ and ~1.1 cm^2^/SV at 300 K, respectively. With a decrease in temperature, these two values increase simultaneously (not shown here), thus giving rise to a prominent reduction in the $\rho_{xx}$ for the studied alloy at its ferromagnetic state (see Figure 1c).

To gain a deeper insight into the mechanism of the AHE for the Co_2_VGa alloy, an inherent relationship between the $\rho_{xx}$ and the anomalous Hall resistivity ($\rho_{\mathrm{AH}}$, indicated by a red solid dot in Figure 3) should be clarified. In principle, there is a direct proportionality between these two physical quantities, which can be described by the relation of $\rho_{\mathrm{AH}}\propto \rho_{xx}^{n}$. Here, the exponent n is a constant, and its value will deliver a vital clue concerning the mechanism of the AHE, that is, if n=1, the extrinsic skew scattering at the impurities is responsible for the AHE; if n=2, both the extrinsic side-jump at the impurities and the intrinsic Berry curvature could account for the AHE [35].

**Figure 4:**
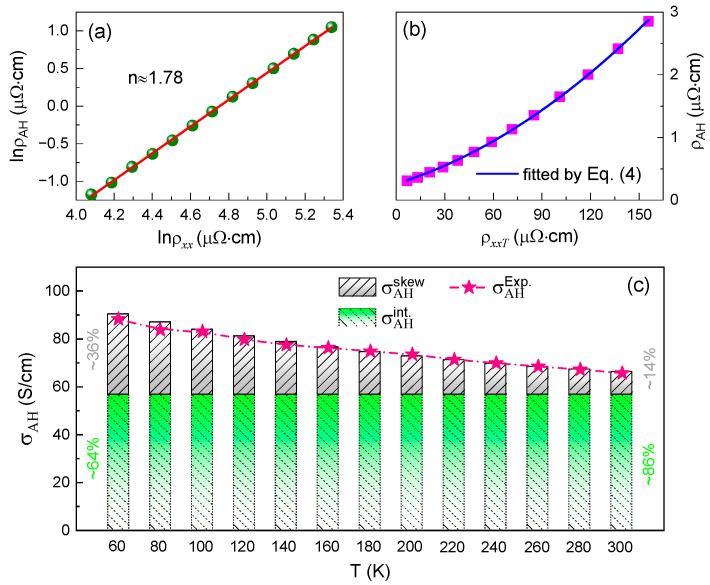
(**a**) The relationship between $\rho_{xx}$ and $\rho_{\mathrm{AH}}$ plotted on a Napierian logarithmic scale, and the red solid line is a linear fitting. (**b**) The $\rho_{\mathrm{AH}}$ as a function of $\rho_{xxT}$, which is fitted by Equation (4). (**c**) The relative contributions between the extrinsic skew scattering ($\sigma_{\mathrm{AH}}^{\mathrm{skew}}$) and the intrinsic Berry curvature ($\sigma_{\mathrm{AH}}^{\mathrm{int}.}$) for total AHC ($\sigma_{\mathrm{AH}}$), and the pink stars indicate the experimental values ($\sigma_{\mathrm{AH}}^{\mathrm{Exp}.}$) which are calculated by the tensor conversion relation of $\rho_{\mathrm{AH}}/\left(\rho_{\mathrm{AH}}^{2}+\rho_{xx}^{2}\right)$.

Figure 4a presents the $\rho_{xx}$ dependence of $\rho_{\mathrm{AH}}$ on a double Napierian logarithmic scale. The exponent n extracted from the slope of the fitting curve was found to be 1.78, which indicates that the AHE for the studied alloy results from the contribution of different mechanisms. To acquire more detailed information associated with the origin of the AHE, Figure 4b further gives $\rho_{\mathrm{AH}}$ as a function of $\rho_{xxT}$, which is fitted by an equation that can be used to exclude the contribution from the residual resistivity for the AHE as follows [36,37]:

(4) $$\rho_{\mathrm{AH}}=\alpha^{\mathrm{skew}}\left(\rho_{0}+\rho_{xxT}\right)+\sigma_{\mathrm{AH}}^{\prime }{\left(\rho_{0}+\rho_{xxT}\right)}^{2}.$$

In the above equation, $\alpha^{\mathrm{skew}}$ is a fitting coefficient connected with the skew scattering, while $\sigma_{\mathrm{AH}}^{\prime }$ corresponds to the AHC arising from the side-jump or intrinsic mechanisms. Both the $\alpha^{\mathrm{skew}}$ and $\sigma_{\mathrm{AH}}^{\prime }$ estimated from the fitting results were found to be 1.99 × 10^−3^ and 56.6 S/cm, respectively. It is apparent that the $\sigma_{\mathrm{AH}}^{\prime }$ is very close to the theoretical value (66 S/cm) of the Co_2_VGa alloy calculated using the Berry curvature in the previous study [38]. Such a small discrepancy probably stems from a tiny difference in the electronic structure between the ideal and nonideal *L*2_1_ structures. In another aspect, the $\sigma_{\mathrm{AH}}^{\prime }$ contributed by the side-jump mechanism can be roughly judged by using the formula of $e^{2}/\left(ha\right)\left(\epsilon_{so}/E_{F}\right)$, where h and a are the Planck constant and the lattice parameter, and $\epsilon_{so}$ and $E_{F}$ are the spin–orbit interaction and the Fermi energy, respectively [39]. Taking the related parameters into the $e^{2}/\left(ha\right)$ term, the calculated value was ~694 S/cm. However, the value of $\epsilon_{so}/E_{F}$ is typically less than an order of $10^{-2}$ for ferromagnetic metals [40,41]. This indicates that the $\sigma_{\mathrm{AH}}^{\prime }$ resulting from the side-jump contribution can be ignored approximately in contrast to the intrinsic mechanism. Because the AHE is mainly governed by the intrinsic Berry curvature and extrinsic skew scattering, the relative contributions of both can be separated in the studied alloy, as shown in Figure 4c. The total AHC ($\sigma_{\mathrm{AH}}$) derived from Equation (4) is consistent with the experimental value ($\sigma_{\mathrm{AH}}^{\mathrm{Exp}.}$, indicated by red stars) calculated using the tensor conversion relation of $\rho_{\mathrm{AH}}/\left(\rho_{\mathrm{AH}}^{2}+\rho_{xx}^{2}\right)$. When the temperature reaches 300 K, the proportion of the intrinsic Berry curvature’s ($\sigma_{\mathrm{AH}}^{\mathrm{int}.}$) contribution to the $\sigma_{\mathrm{AH}}$ arrives at ~86%. When the temperature decreases from 300 to 60 K, it can be found that the skew scattering contribution to the $\sigma_{\mathrm{AH}}$ increases from ~10 to ~32 S/cm. A change of this magnitude is higher than that reported in the Co_2_FeGe [22] and Co_2_FeAl [23] alloys. Considering that the resistivity contributed by the $T^{2}$ term signally overmatches that contributed by the $T^{9/2}$ and T terms within the measured temperature range (see Figure 2b), this behavior could be interpreted as the enhancement of the effective spin–orbit interaction of the electron with a decreased temperature [35]. Moreover, departing from the relation of tensor conversion, a decrease in longitudinal resistivity with decreasing temperature can also increase the value of $\sigma_{\mathrm{AH}}$ contributed from the skew scattering. Despite this fact, the $\sigma_{\mathrm{AH}}^{\mathrm{int}.}$ still accounts for 64% of the total contribution at 60 K, which demonstrates that the intrinsic Berry curvature plays a crucial role in the AHE of the Co_2_vGa Heusler alloy.

## 4. Conclusions

In summary, the structural, magnetic, and transport properties of the Co_2_vGa half-metallic Heusler alloy were systematically studied. Structural and magnetic measurements make clear that the studied alloy possesses a relatively high-order *L*2_1_ cubic phase at room temperature and undergoes a Curie transition from a paramagnetic state to a ferromagnetic state at ~350 K. In the temperature range from 30 to 75 K, the electrical transport measurement suggests that the resistivity follows the $T^{9/2}$ dependence related to two-magnon scattering, which provides a signature of the half-metallic nature of the studied alloy. At the same time, the half-metallicity of this alloy was also confirmed by the reverse sign of magnetoresistance around 100 K. Furthermore, magnetic transport measurements indicated the presence of a relatively high $R_{AH}$ at 300 K for the studied alloy. A careful analysis shows that the origin of the AHE for the studied alloy is induced by extrinsic skew scattering and the intrinsic Berry curvature, but the latter contribution dominates over the former one. These experimental studies provide an additional insight into the fundamental characteristics of the Co_2_VGa half-metallic ferromagnetic Heusler alloy.

## Data Availability

The data presented in this study are available on request from the corresponding author.
